# Compulsive versifying after treatment of transient epileptic amnesia

**DOI:** 10.1080/13554794.2014.953178

**Published:** 2014-10-26

**Authors:** Ione O. C. Woollacott, Phillip D. Fletcher, Luke A. Massey, Amirtha Pasupathy, Martin N. Rossor, Diana Caine, Jonathan D. Rohrer, Jason D. Warren

**Affiliations:** ^a^ Dementia Research Centre, Department of Neurodegenerative Disease, UCL Institute of Neurology, University College London, London, UK; ^b^ Specialist Mental Health Team for Older People, The Meadows, Hertfordshire Partnership NHS Foundation Trust, Hertfordshire, UK; ^c^ Department of Neuropsychology, National Hospital for Neurology and Neurosurgery, London, UK

**Keywords:** hypergraphia, verbal creativity, poetry, transient epileptic amnesia, lamotrigine, temporal lobe, dementia

## Abstract

Compulsive production of verse is an unusual form of hypergraphia that has been reported mainly in patients with right temporal lobe seizures. We present a patient with transient epileptic amnesia and a left temporal seizure focus, who developed isolated compulsive versifying, producing multiple rhyming poems, following seizure cessation induced by lamotrigine. Functional neuroimaging studies in the healthy brain implicate left frontotemporal areas in generating novel verbal output and rhyme, while dysregulation of neocortical and limbic regions occurs in temporal lobe epilepsy. This case complements previous observations of emergence of altered behavior with reduced seizure frequency in patients with temporal lobe epilepsy. Such cases suggest that reduced seizure frequency has the potential not only to stabilize or improve memory function, but also to trigger complex, specific behavioral alterations.

The neural substrates of complex behaviors remain largely unknown and this is especially true of creative activities such as music and poetry (Zeman, Milton, Smith, & Rylance, 2013). In contrast to the functional deficits commonly associated with brain damage, certain brain disorders produce excessive, abnormal, and sometimes highly specific behavioral alterations. Such disorders may hold insights into the brain mechanisms that mediate these behaviors. Examples include pathological gambling, punding, and hypersexuality associated with dopaminergic replacement in Parkinson's disease (Joutsa, Martikainen, & Kaasinen, 2012), hyper-religiosity associated with focal temporal lobe atrophy (Chan et al., 2009), heightened visual creativity and production of art in frontotemporal dementia (Miller et al., 1998; Seeley et al., 2008), and musicophilia associated with degenerative and epileptic processes involving the mesial temporal lobes (Fletcher, Downey, Witoonpanich, & Warren, 2013; Rohrer, Smith, & Warren, 2006). Compulsive writing behavior, or hypergraphia, has also been associated with epilepsy and other disorders, such as tumors or stroke, affecting the temporal lobes and non-dominant hemisphere (Flaherty, 2005; Imamura, Yamadori, & Tsuburaya, 1992; Kalamangalam, 2009; Mendez, 2005; Waxman & Geschwind, 2005; Yamadori, Mori, Tabuchi, Kudo, & Mitani, 1986).

Transient epileptic amnesia (TEA) is a distinctive syndrome of temporal lobe epilepsy often accompanied by a persistent interictal disturbance of memory (Butler et al., 2007, 2009; Zeman, Boniface, & Hodges, 1998; Zeman & Butler, 2010); proposed diagnostic criteria include a history of recurrent witnessed episodes of transient amnesia, intact cognitive functions other than memory during typical episodes, and other evidence for a diagnosis of epilepsy (Zeman et al., 1998). While a substantial proportion of patients with TEA have interictal impairment of remote autobiographical memory, accelerated long-term forgetting and deficits of topographical memory and spatial navigation (Butler et al., 2007), extramnestic cognitive deficits or significant behavioral disturbance are not commonly reported. Here we describe the case of a patient with TEA who developed a highly specific and “creative” form of hypergraphia – compulsive versifying – after commencement of anticonvulsant therapy.

## Clinical details

A 76-year-old right-handed woman presented with a 5-year history of progressive impairment of episodic and topographical memory, manifesting as increasing forgetfulness for both recent and remote lifetime events and a tendency to lose her way even in familiar locations. Her husband reported that for the last two years, she had been having recurrent episodes, occurring up to several times weekly, in which she would stare and become unresponsive, associated with mouthing and chewing movements. These episodes typically lasted up to a minute and were of sudden onset with rapid recovery of awareness but ensuing repetitive questioning about where she was and what was happening, lasting around an hour. The episodes frequently occurred on waking, but could occur at any time of day. She had no memory of these episodes subsequently. Going into the history in more detail, the patient and her husband had the impression that she sometimes seemed able to retain new information initially, but this was later more or less completely lost over a period of days to weeks. There was no history of cognitive complaints beyond the domains of memory, no other neurological symptoms and no known history of seizures or other neurological or psychiatric disorders. Her husband confirmed that there had been no disturbance of her personality or social behavior. Her past medical history included atrial fibrillation, hypertension and aortic stenosis, treated with warfarin, digoxin, perindopril, bisoprolol, verapamil, and a cardiac pacemaker. Her maternal grandmother had developed dementia; no details were available and there was no other relevant family history. On bedside cognitive examination she scored 24/30 on the Mini-Mental State Examination (MMSE), losing points for orientation and recall; she had a well-preserved social façade but evidence of impaired recall and recognition and word finding difficulty. This was corroborated by detailed neuropsychological assessment (summarized in Table 1) demonstrating superior verbal and average performance IQ, but profoundly impaired recognition and recall of verbal and visual information, and additional deficits of face naming and recognition and visual object apperception. Other executive and language skills were well preserved. The general neurological examination was normal.

**Table 1.** Results of neuropsychological assessment in this case.

Neuropsychological test	Score	Norms
**General intellectual functions – WAIS-III**		
Verbal IQ	125	Superior
Vocabulary	55	Superior
Digit span	23	Very superior
Similarities	24	High average
Performance IQ	102	Average
Picture completion	16	Average
Matrix reasoning	10	Average
Block design	24	Average
**Pre-morbid functioning estimate**		
NART IQ	126	Superior
**Episodic memory**		
Short Recognition Memory Test Words (25)	**15**	**Impaired (<5th)**
Short Recognition Memory Test Faces (25)	**14**	**Impaired (<5th)**
AIMPB story		
Immediate recall	8	Low average (<10th)
Delayed recall	0	Low average (<10th)
AIMPB figure		
Copy	75	Average (25th)
Delayed recall	**0**	**Impaired (<10th)**
**Language and semantic memory**		
Graded Naming Test (30)	18	Average (25–50th)
Concrete Word Synonym Test (25)	24	High average (75–90th)
Famous faces recognition (12)	**1**	**Impaired (<5th)**
**Visual perception**		
VOSP: object decision (20)	**9**	**Impaired (<5th)**
VOSP: cube analysis (10)	9	Average (>5th)
Graded Spelling Test (30)	29	Superior (>95th)
Graded Difficulty Calculation Test (24)	18	Superior (>95th)
**Executive functions**		
Fluency		
Semantic fluency (“animals”)	19	High average (75th)
Phonemic fluency (“S”)	17	Average (25–50th)
Stroop Color Word Test		
Color (time)	26 s	
Color – word (112)	69	Low average (14–16th)
Weigl (2)	2	Average
**Speed and attention**		
Canceling O’s	56	Low average (76th)

Notes: Summary of the patient’s performance on clinical neuropsychological assessment prior to clinical seizure cessation and treatment with lamotrigine. Numerals in first column (in parentheses) indicate maximum test scores; deficits referenced to normative data are indicated in bold (percentiles are shown in parentheses in last column). AIMPB, Adult Memory and Information Processing Battery; IQ, Intelligence Quotient; NART, National Adult Reading Test; VOSP, Visual Object and Space Perception; WAIS-III, Wechsler Adult Intelligence Scale III.

**Figure 1.**
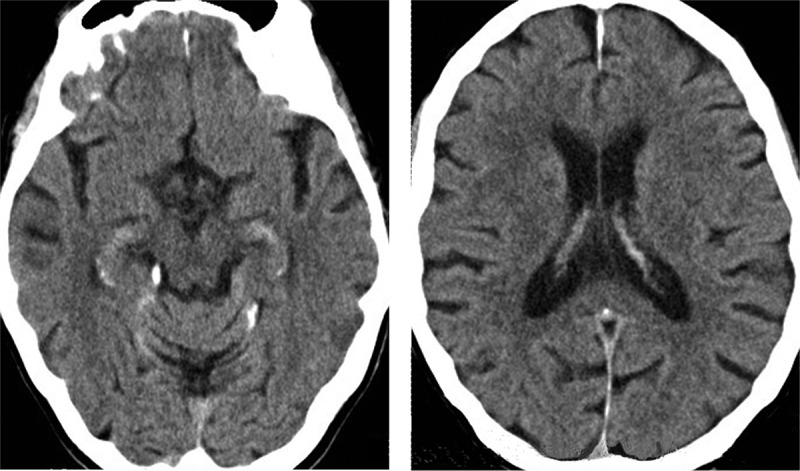
Computed tomography axial sections of the patient’s brain, at the level of the hippocampi (left panel) and inferior parietal lobes (right panel); the left hemisphere is shown on the right side of the image in both sections.

The patient underwent a number of investigations. Routine blood screens were unremarkable. Brain CT (MRI contraindicated) showed mild diffuse cerebral volume loss without disproportionate temporal lobe atrophy, and mild white matter ischemic changes (Figure 1). Routine EEG revealed a preserved alpha rhythm and interictal left anterior temporal sharp and slow waves. In light of these findings and the clinical picture including prominent amnestic episodes, the patient received a primary diagnosis of TEA; however, it was felt that an additional, underlying neurodegenerative process (in particular, Alzheimer's disease) could not be firmly excluded. An initial trial of levetiracetam (to 1500 mg twice daily) and addition of donepezil (to 10 mg daily) did not ameliorate her cognitive complaints, nor her amnestic episodes and temporal lobe seizures. However, these episodes and clinical seizures immediately and completely ceased with addition of a low dose of lamotrigine (25 mg daily); levetiracetam was subsequently withdrawn. Her cognitive profile remained stable over an 18-month follow-up interval and MMSE was 26/30 at the last follow-up. Subjective memory difficulties were improved, such that she had better recall of recent events following seizure cessation and appeared to forget new information less rapidly. However, she still described difficulty recalling more remote episodic memories. Repeat EEG several months following seizure cessation showed no evidence of epileptiform activity or other abnormalities.

**Figure 2.**
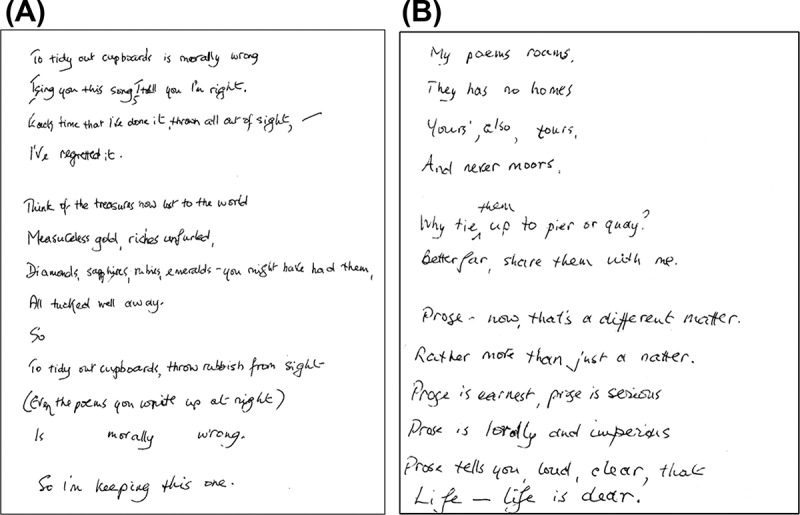
Examples of verse produced by the patient (transcriptions follow). (A) To tidy out cupboards is morally wrong / I sing you this song, I tell you I’m right. / Each time that I’ve done it, thrown all out of sight, / I’ve regretted it. // Think of the treasures now lost to the world / Measureless gold, riches unfurled, / Diamonds, sapphires, rubies, emeralds – you must have had them, / All tucked well away. / So // To tidy out cupboards, throw rubbish from sight / (Even the poems you write up at night) / Is morally wrong. / So I’m keeping this one. (B) My poems roams, / They has no homes / Yours’, also, tours, / And never moors. // Why tie them up to pier or quay? / Better far, share them with me. // Prose – now, that’s a different matter. / Rather more than just a natter. / Prose is earnest, prose is serious / Prose is lordly and imperious / Prose tells you, loud, clear, that / Life – life is dear.

Several months after starting lamotrigine, the patient suddenly began to write original verse. Whereas poetry had never previously been among her pastimes, she now produced copious short poems (around 10–15 each day) on quotidian topics such as housework or about the act of versifying itself and sometimes expressing her opinions or regret about past events. These poems often had a wistful or pessimistic nature but did not have a moral or religious focus. Her husband characterized them as “doggerel” because they were generally rhyming and often featured puns and other wordplay (examples in Figure 2). This versifying had a compulsive quality: she spent several hours per day writing poetry and became irritated if attempts were made to disengage her. However, she appeared to derive pleasure from the activity and there was no evidence of associated distress. She did not produce prose passages, diaries, or other examples of hypergraphia, nor did she develop new interests in other “creative media,” such as visual arts or music. When reassessed 6 months after the onset of versifying this apparent compulsion had diminished, but she continued to produce occasional poems. She had also developed a more general fondness for wordplay, frequently using puns in speech, making humorous word associations, and identifying word patterns in everyday objects such as car license plates. Throughout this period there were no associated mood symptoms, features of a thought disorder, or other changes in her behavior or cognition to suggest hypomania or another generalized neuropsychiatric disturbance.

## Discussion

This case illustrates an unusual behavioral alteration in an unusual clinical context. Although hypergraphia is well described in the setting of epilepsy and other disorders affecting the temporal lobes and non-dominant hemisphere (Flaherty, 2005; Imamura et al., 1992; Kalamangalam, 2009; Mendez, 2005; Waxman & Geschwind, 2005; Yamadori et al., 1986), hypergraphic output is typically rambling or disorganized and often consists of extensive lists, memory aids, or diary entries (Flaherty, 2005; Waxman & Geschwind, 2005). In contrast, our patient's versifying took the form of discrete, highly structured, creative outputs (poems). She lacked the hypomanic and other behavioral disturbances that commonly accompany hypergraphia (Flaherty, 2005) and, in contrast to previously reported cases as detailed in Waxman and Geschwind (2005), her verse tended to be based on everyday experiences or commentaries.

Hypergraphia has been associated with right temporal lobe seizure foci, putatively due to disinhibition of left temporal lobe language centers, and generally arises interictally in the context of ongoing seizures (Flaherty, 2005; Kalamangalam, 2009; Mendez, 2005; Waxman & Geschwind, 2005). However, in the present case, EEG evidence pointed to a probable seizure focus in the left temporal lobe and versifying manifested only after seizures clinically and electrographically diminished following anticonvulsant therapy. Heightened or compulsive production of verse has been described rarely in association with right hemisphere seizure foci (Mendez, 2005; Waxman & Geschwind, 2005), but may also develop in other disorders. Patients with Parkinson's disease may produce poetry in the context of other compulsive behaviors consistent with enhanced dopaminergic drive (Joutsa et al., 2012). Patients with semantic variant primary progressive aphasia may engage in copious wordplay, including poetry and prose (Wu et al., 2013). Previously reported cases have had predominant bilateral anterior temporal lobe atrophy with marked involvement of mesial temporal structures, but relative sparing of dorsolateral temporal cortices and classical language areas.

The syndromic diagnosis in this case was not entirely straightforward. On the one hand, our patient exhibited a number of features in line with previous descriptions of TEA. These included the phenomenology of her attacks and ensuing amnesic periods, sparing of cognitive domains such as executive function and language, EEG findings and compelling response to low dose anticonvulsant monotherapy (Butler et al, 2007). The syndromic diagnosis of TEA in this case was further corroborated by the pervasive impairment of both retrograde and anterograde autobiographical memory (Butler et al., 2007, 2009; Manes, Graham, Zeman, de Lujan, & Hodges, 2005; Milton et al., 2010) and (though not formally substantiated) the historical suggestion of accelerated forgetting (Butler et al., 2007, 2009; Manes et al., 2005). The presence of clinical features of temporal lobe seizures, the initial neuropsychological profile with interictal impairment of anterograde episodic memory, the rather inconclusive neuroimaging study, and the persistence of residual memory dysfunction on anticonvulsant therapy are also all in keeping with a diagnosis of TEA (Butler et al., 2007; Milton et al., 2010). On the other hand, our patient developed amnestic episodes late in life with a long reported prodrome of memory decline prior to onset of the typical episodes, raising the possibility of an underlying neurodegenerative process. This patient did not fulfil diagnostic criteria for a parkinsonian disorder or semantic variant primary progressive aphasia (neurodegenerative syndromes previously linked to hypergraphia: Joutsa et al., 2012; Wu et al., 2013), nor did she exhibit the pervasive behavioral disturbances and social disintegration that characterize the behavioral variant of frontotemporal dementia (Warren, Rohrer, & Rossor, 2013). Moreover, there was no selective mesial temporal lobe or other focal atrophy on brain CT in this case, though detailed assessment of the relevant brain regions (requiring MRI) was not possible. On balance, however, underlying Alzheimer's disease pathology remains possible, and only a longer period of observation will resolve this: the presence of additional cognitive deficits in domains of face and visuoperceptual processing (Table 1) may be a clue to this possibility, though dysfunctional bi-temporal mechanisms could conceivably provide a unifying neuroanatomical substrate for these deficits. The chronological relationship of our patient's versifying to seizure treatment argues against evolving neurodegeneration as the sole basis for her selective behavioral disturbance. Of further note, hypergraphia has not, to our knowledge, been reported previously as a manifestation of Alzheimer's disease. It is, however, plausible that Alzheimer's or other underlying neurodegenerative pathology might facilitate the emergence of such behavior, interacting with the effects of brain network disruption due to temporal lobe seizures.

A further unresolved dimension in this case is the role played by anticonvulsant therapy. Our patient was taking a number of medications when compulsive versifying emerged. Hypomania has been described previously in association with lamotrigine (Villari, Rocca, Frieri, & Bogetto, 2008) and rarely with donepezil (Wicklund & Wright, 2012), but neither of these medications has been reported to cause isolated hypergraphia, and our patient had no features of hypomania. While we cannot rule out entirely the possibility that her versifying was a side effect of drug treatment per se, the present case may be analogous with the previously reported case of a patient with TEA who developed a specific craving for music after cessation of seizures on lamotrigine (Rohrer et al., 2006). Such specific behavioral disturbances might arise via the interacting effects of seizure amelioration and the pharmacological properties of lamotrigine. Despite the very low dose of lamotrigine used here, reduction in clinical seizure frequency was accompanied by electroencephalographic improvement, suggesting that seizures had abated rather than merely transformed. Chronic seizure activity has been proposed to lead to a reorganization of dominant temporo-limbic circuitry, linking semantic and other linguistic mechanisms with the limbic machinery of drives and rewards (Flaherty, 2005; Rohrer et al., 2006). In our patient, improved seizure control could theoretically have restored activity in these reorganized circuits. The development of specific behavioral disturbances following seizure reduction might lie on a continuum with emergent psychosis or “forced normalization,” previously described in association with successful treatment of temporal lobe epilepsy with various anticonvulsants, including lamotrigine (Clemens, 2005).

The nature of our patient's behavioral change raises a broader issue concerning the brain mechanisms that sustain “creativity.” Novel idea generation has been modelled as a reciprocal interaction of fronto-temporo-limbic networks modulated by dopaminergic and other neurotransmitter pathways (Flaherty, 2005), while functional neuroimaging studies in the healthy brain have shown that generation or appreciation of rhyme and other novel verbal output engages distributed bi-hemipsheric peri-Sylvian and antero-mesial temporal lobe networks (Kareken, Lowe, Chen, Lurito, & Mathews, 2000; Shah et al., 2013; Zeman et al., 2013). While the potential of brain damage to erode the creative process is therefore obvious, it has recently been proposed that certain brain disorders (notably frontotemporal dementia) may exceptionally preserve or enhance creativity across a variety of different media (Flaherty, 2005; Fletcher et al., 2013; Miller et al., 1998; Seeley et al., 2008; Wu et al., 2013). Whereas objective assessment of the quality of creative output is notoriously difficult, the drive to create may be easier to index (for example, by quantity of output or time spent engaged in the activity) in patients with neurological disease. To determine the brain basis for abnormal, creative behaviors will require detailed neuroanatomical correlation, which was not possible in the present case. Notwithstanding the neuroanatomical caveat, this case complements previous descriptions of altered creative behavior in patients with temporal lobe epilepsy and neurodegenerative disease, and further suggests that reduced seizure frequency has the potential not only to stabilize or improve memory function but also to trigger complex, specific behavioral routines.

## Disclosure statement

JDW receives salary support from the Wellcome Trust. The remaining authors have no competing interests.
